# Construction of Chitosan-Modified Naphthalimide Fluorescence Probe for Selective Detection of Cu^2+^

**DOI:** 10.3390/s24113425

**Published:** 2024-05-26

**Authors:** Chunwei Yu, Jin Huang, Mei Yang, Jun Zhang

**Affiliations:** NHC Key Laboratory of Tropical Disease Control, School of Tropical Medicine, Hainan Medical University, Haikou 571199, China; hy0211049@hainmc.edu.cn (C.Y.); abchuangj@163.com (J.H.); yang24364@hainmc.edu.cn (M.Y.)

**Keywords:** Cu^2+^, chitosan, 1, 8-naphthalimide, fluorescence

## Abstract

A chitosan-based Cu^2+^ fluorescent probe was designed and synthesized independently using the C-2-amino group of chitosan with 1, 8-naphthalimide derivatives. A series of experiments were conducted to characterize the optical properties of the grafted probe. The fluorescence quenching effect was investigated based on the interactions between the probe and common metals. It was found that the proposed probe displayed selective interaction with Cu^2+^ over other metal ions and anions, reaching equilibrium within 5 min.

## 1. Introduction

Heavy metal ions are important environmental pollutants, primarily caused by human activities. The disruption caused by heavy metal ions can lead to their accumulation and even transmission in plants and animals, from lower to higher levels in the food chain, resulting in an extreme ecotoxicological impact, particularly on humans [1,2,3]. Cu^2+^ is a significant environmental pollutant and a harmful element in biological systems under overload conditions, which may lead to neurodegenerative disorders due to its likely involvement in the formation of reactive oxygen species [4,5,6]. The detection of Cu^2+^ in vitro or in vivo consistently represents an active area of research.

Various traditional analytical methods exist, including AAS (atom absorption spectrometry), ICP-ES (inductively coupled plasma emission spectrometry) and ICP-MS (inductively coupled plasma mass spectrometry), etc. It is well known that these technologies offer good intelligent automation and limits of detection, but are very expensive and do not easily analyze on site [7,8,9]. Fluorescence probes have become a promising strategy because of their simplicity, non-destructive characteristics and structural modification to various conditions, etc. [10,11,12], which can translate molecular recognition information into tangible fluorescence signals by connecting a specific group to a fluorophore through organic methods. Currently, the development of Cu^2+^-specific fluorescent probes has been extensively explored [13,14,15]. Even though many innovative achievements have been made to realize Cu^2+^-related detection, most of them restricted their applications due to a long equilibrium time, lack of selectivity or poor sensitivity [16,17,18]. Therefore, the development of new technologies is urgently needed to address these issues to meet a wider range of demands. In recent years, researchers have explored a novel approach to design and prepare sensing materials by modifying chitosan with fluorescence dyes. These fluorescent materials have been applied to recognize heavy metal ions and have shown high selectivity and sensitivity.

Chitosan, the only cationic natural polysaccharide found in nature so far, has been widely used in cosmetics, the food industry, medical supplies and bioengineering, etc. [19,20,21]. Firstly, chitosan is a natural polymer with non-toxic, hydrophilic properties, biodegradability, and good renewability. Secondly, the hydroxyl and amino groups within chitosan molecules exhibit strong activity, making them easily amenable to chemical grafting and modification. Additionally, the presence of amino and hydroxyl groups enables chitosan molecules to form intramolecular and intermolecular hydrogen bonds, resulting in a three-dimensional complex network structure capable of chelating heavy metal ions. Lastly, the attachment of multiple dye molecules onto a single chitosan molecule exhibits an optical additive effect during tissue binding and imaging, which significantly enhances the sensitivity and reduces the usage of probe. These unique properties render chitosan as an ideal carrier for functional fluorescent probes. In recent years, the amino group has been identified as the key factor affecting the activity of chitosan in the construction of fluorescent probes. Pournaki constructed a fluorescent probe through the esterylamolysis reaction between C-2 amino of chitosan and benzopyran derivatives to realize the identification of Fe^3+^ under acidic conditions [22]. Men et al. produced a Schiff base using the chitosan C-2-amino group with rhodamine glyoxal derivatives, which was successfully applied to recognize and adsorb Hg^2+^ in water [23]. The aforementioned studies validated the potential application of chitosan-based multifunctional materials in metal ion analysis.

**Figure 1:**
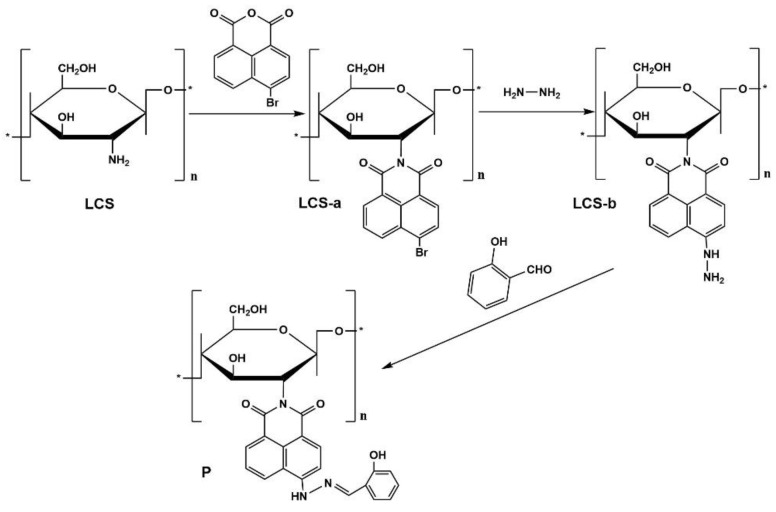
Synthetic scheme for compound **P**, “*” represents the repeated units.

Herein, we presented a novel fluorescent material obtained through the modification of chitosan with naphthalimide dye. We anticipated that this fluorescent material could serve as a probe for the analysis of Cu^2+^ in environmental samples through fluorimetric or colorimetric methods. The synthetic scheme employed for chitosan-based fluorescent materials is illustrated in Figure 1.

## 2. Materials and Methods

### 2.1. Instruments and Reagents

Fluorescence spectra were determined with an F-4600 fluorescence spectrometer (Hitachi, Tokyo, Japan). FT-IR spectra were recorded on a Nicolet Magna-IR 750 spectrometer equipped with a Nic-Plan Microscope (Nicolet, Madison, WI, USA). ^1^H NMR spectra were performed by a Bruker AV 400 instrument with tetramethylsilane (TMS) as an internal standard and DMSO-*d_6_* as a deuterium generation reagent (Bruker, Karlsruche, Germany). Absorption spectra were measured with a U-2910 spectrophotometer (Hitachi, Tokyo, Japan). All pH measurements were made with a Model PHS-3C meter (Jinpeng, Shanghai, China).

All reagents were analytically pure and purchased from Sigma-Aldrich Co. (St. Louis, MO, USA) without special treatment before use. The metal ion salts used were NaCl, KCl, CaCl_2_·2H_2_O, MgCl_2_·6H_2_O, CdCl_2_, CrCl_3_·6H_2_O, HgCl_2_, CuCl_2_·2H_2_O, FeCl_3_·6H_2_O, AgNO_3_ and AlCl_3_·6H_2_O; and anion species were from various salts such as NaHCO_3_, NaNO_3_, Na_2_CO_3_, NaF, Na_2_SO_4_, Na_2_C_2_O_4_ and Na_2_HPO_4_.

### 2.2. Synthesis of LCS-a

First, 1.0013 g of chitosan (LCS) was dissolved in 200 mL of anhydrous ethanol. Subsequently, 3.0185 g of 4-bromo-1, 8-naphthalene anhydride was added to the reaction flask. The mixture was refluxed for 8 h. The resulting product was then promptly filtered and washed with hot ethanol. The precipitate of LCS-a obtained was subjected to extraction using Sechelt’s extractor with ethanol for 12 h. IR (KBr): 3425.23 cm^−1^, 1779.22 cm^−1^, 1732.91 cm^−1^, 1588.16 cm^−1^, 1570.09 cm^−1^, 1299.06 cm^−1^, 1224.03 cm^−1^, 1132.76 cm^−1^, 1022.02 cm^−1^, 773.77 cm^−1^.

### 2.3. Synthesis of LCS-b

First, 0.5000 g of newly prepared LCS-a, 20 mL of anhydrous ethanol and 8 mL of hydrazine hydrate (85%) were placed in a round-bottom flask. The reaction mixture was refluxed for 6 h and cooled to room temperature. Then, the red brown precipitate of LCS-b was extracted by Sechelt’s extractor with ethanol for at least 12 h. IR (KBr): 3441.09 cm^−1^, 3329.42 cm^−1^, 1638.30 cm^−1^, 1581.41 cm^−1^, 1537.33 cm^−1^, 1396.95 cm^−1^, 1366.06 cm^−1^, 1254.23 cm^−1^, 974.48 cm^−1^, 768.30 cm^−1^.

### 2.4. Synthesis of **P**

First, 0.2500 g of LCS-b and 40 mL of anhydrous ethanol were put into a three-neck flask. Then, 1.1 mL of salicylaldehyde was added dropwise. After reflux for 7 h, it was cooled to room temperature and filtered to obtain the crude product, which was further purified using a Soxhlet extractor with anhydrous ethanol for 4 h to obtain **P**. IR (KBr): 3434.04 cm^−1^, 3278.44 cm^−1^, 1666.57 cm^−1^, 1583.60 cm^−1^, 1382.21 cm^−1^, 1336.13 cm^−1^, 1290.53 cm^−1^, 1239.53 cm^−1^, 1129.35 cm^−1^, 757.91 cm^−1^.

### 2.5. Preparation of the Test Solution

The solutions of various testing metal ion species were prepared from NaCl, KCl, CaCl_2_·2H_2_O, MgCl_2_·6H_2_O, CdCl_2_, CrCl_3_·6H_2_O, HgCl_2_, CuCl_2_·2H_2_O, FeCl_3_·6H_2_O, AgNO_3_ and AlCl_3_·6H_2_O; and anion species were from various salts such as NaHCO_3_, NaNO_3_, Na_2_CO_3_, NaF, Na_2_SO_4_, Na_2_C_2_O_4_ and Na_2_HPO_4_ to obtain 1 mM stock solution in the twice-distilled water. A solution containing 2000 ppm of **P** was prepared in DMSO.

### 2.6. UV–Vis and Fluorescence Titration

Test solutions were prepared by placing 50 μL of the **P** stock solution (2000 ppm) into a test tube, adding an appropriate aliquot of individual ions stock solution (1 mM), and then diluting the solution to 5 mL with aqueous-ethanol media (pH 7.0, 20 mM HEPES, *v*:*v* = 1:9). The excitation wavelength was recorded at 430 nm. The test medium was in the aqueous-ethanol media (pH 7.0, 20 mM HEPES, *v*:*v* = 1:9).

### 2.7. The Calculation of the Combined Constant

The binding constants for the formation of the **P**-Cu^2+^ complex were evaluated using the Benesi–Hildebrand plot [24].

$$\frac{1}{F-F_{0}}=\frac{1}{K(F_{\max}-F_{0}){\left[Cu^{2+}\right]}_{0}^{n}}+\frac{1}{F_{\max}-F_{0}}$$

*F*_0_ is the fluorescence intensity of **P** without Cu^2+^, *F* is the fluorescence intensity of **P** obtained with Cu^2+^, *F*_max_ is the fluorescence intensity of **P** in the presence of an excess amount of Cu^2+^ and *K* is the binding constant (M^−1^) determined from the slope of the linear plot.

## 3. Results and Discussion

### 3.1. FTIR and ^1^H NMR Spectra of the LCS, LCS-a and LCS-b

**Figure 2:**
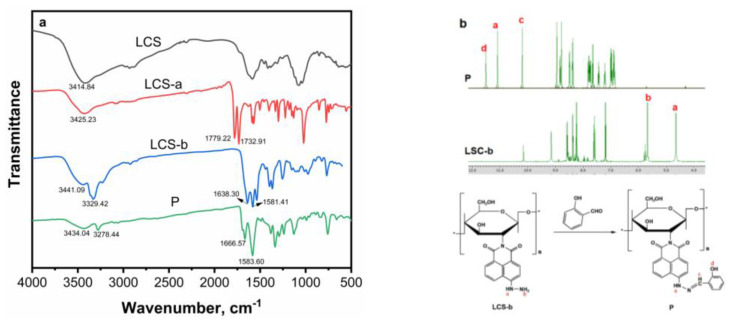
(**a**) FTIR pattern of LCS, LCS-a, LCS-b and **P**; (**b**) ^1^H NMR of LCS-b and **P**, “a–d” stand for H form -NH, -NH_2_, -N=CH- and -OH groups, respectively; “*” represents the repeated units.

FTIR spectroscopy was employed to ascertain that the grafting of the naphthalimide derivative onto chitosan was successful. As illustrated in Figure 2a, the IR spectra of LCS, LCS-a, LCS-b and **P** were presented. Initially, LCS exhibited vibrations about ~3400 cm^−1^ corresponding to the hydroxyl groups. Subsequent to the reaction of chitosan with naphthalimide, the characteristic absorption peak of the carbonyl group emerged in the range of 1800–1700 cm^−1^, signifying the successful synthesis of LCS-a. Following the grafting of the hydrazyl group, LCS-b exhibited an absorption peak of the carbonyl group at 1600 cm^−1^, accompanied by increased conjugation, while the characteristic absorption peak of -NH_2_ appeared at 3300 cm^−1^. After the Schiff base condensation reaction between LCS-b and salicylaldehyde, the strong absorption peak at 1583 cm^−1^ was attributed to the stretching vibration of C=N and the skeleton vibration of the benzene ring, and the absorption peak of -NH_2_ disappeared. Crucially, a shift in the carbonyl stretching (i.e., 1666 cm^−1^) of the grafted chitosan was observed, which was attributed to the presence of hydrogen bonding, possibly between the carbonyl oxygen of naphthalimide and the available hydroxyl and unreacted amine groups of chitosan. These results collectively indicated the successful construction of **P**.

In ^1^H NMR spectra of LSC-b (Figure S1) and **P** (Figure S2), the characteristic peak of -NH_2_ at 5.67 ppm disappeared after the reaction of LCS-b and salicylaldehyde. The peak of -NH shifted from a high field of 4.64 ppm to a low field of 11.10 ppm, while the characteristic peaks of -OH and -N=CH appeared at 11.52 and 10.20 ppm, respectively, which also confirmed the formation of **P**.

### 3.2. Application of **P** for the Detection of Cu^2+^

**Figure 3:**
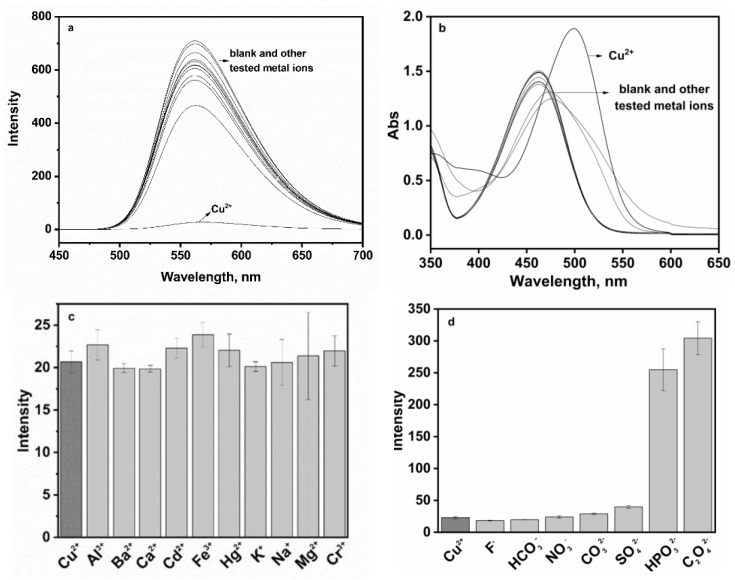
(**a**) Selectivity of **P** (20 ppm) in the presence of common metal ions (100 µM) including Na^+^, K^+^, Ca^2+^, Mg^2+^, Cd^2+^, Hg^2+^, Ag^+^, Cr^3+^, Fe^3+^ and Al^3+^; (**b**) absorption spectra of **P** (20 ppm) for metal ions (100 µM); (**c**) fluorescence response of **P** (20 ppm) to Cu^2+^ (100 µM) in the presence of other metal ions (100 µM); (**d**) fluorescence response of **P** (20 ppm) to Cu^2+^ (100 µM) in the presence of anion ions including HCO_3_^−^, NO_3_^−^, CO_3_^2−^, F^−^, SO_4_^2−^, C_2_O_4_^2−^ and HPO_4_^2−^ (100 µM) in the aqueous-ethanol media (pH 7.0, 20 mM HEPES, *v*:*v* = 1:9).

Fluorescent material **P** featured excellent optical property and displayed strong green fluorescence at 560 nm in 10% aqueous solution at pH 7.0 (Figure 3a). With 100 µM of Cu^2+^, the fluorescence intensity of **P** (20 ppm) was almost completely quenched, which could be ascribed to a PET mechanism and/or a paramagnetic effect of Cu^2+^ [25]. No significant spectral changes in **P** occurred in the presence of alkali or alkaline, earth metals or the first-row transition metals including Na^+^, K^+^, Ca^2+^, Mg^2+^, Cd^2+^, Hg^2+^, Ag^+^, Cr^3+^, Fe^3+^ and Al^3+^. The absorption spectra of **P** to Cu^2+^ were depicted in Figure 3b. **P** displayed an absorption band with a peak at 462 nm, which was attributed to the energy bond of *n*-π* and π-π* in the electron transition of naphthalimide. Upon addition of Cu^2+^, there was a noticeable spectral change accompanied by a red-shifted absorption peak at 499 nm. Furthermore, upon adding 1 equiv. of Cu^2+^ to the above other metal ions solution (100 μM), drastic quenching occurred, consistent with the addition of 1 equiv. of Cu^2+^ alone, indicating that Cu^2+^-specific responses were not affected by competitive metal ions (Figure 3c). The fluorescence responses of probe **P** to Cu^2+^ in the presence of various coexistent anions such as HCO_3_^−^, NO_3_^−^, CO_3_^2−^, F^−^, SO_4_^2−^, C_2_O_4_^2−^ and HPO_4_^2−^ were also investigated. However, it is worth noting that C_2_O_4_^2−^ and HPO_4_^2−^ caused some interference.

**Figure 4:**
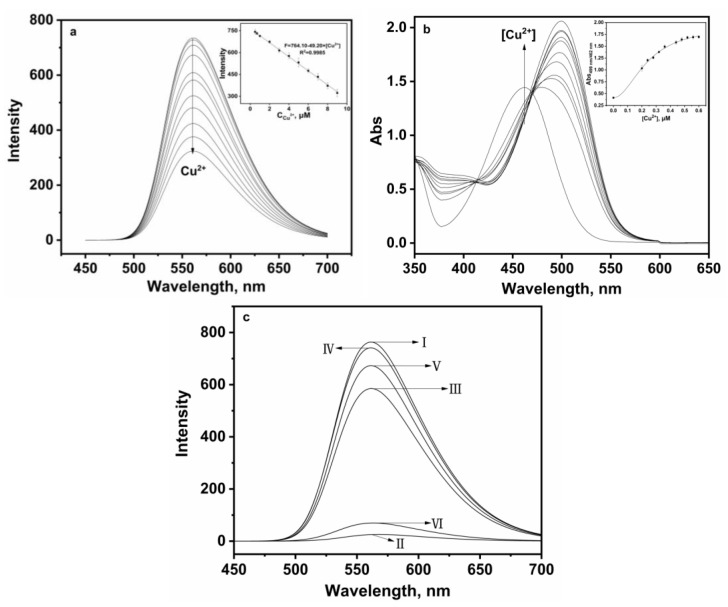
(**a**) Fluorescence spectra of **P** (20 ppm) in the presence of different amounts of Cu^2+^ (0.5–9 μM) in the aqueous-ethanol media (pH 7.0, 20 mM HEPES, *v*:*v* = 1:9). Inset: Linear fluorescence intensity at 561 nm of **P** (20 ppm) upon addition of Cu^2+^ (0.5–9 μM); (**b**) absorption spectra of **P** (20 ppm) in the presence of different amounts of Cu^2+^ (0–0.6 μM) in the aqueous-ethanol media (pH 7.0, 20 mM HEPES, *v*:*v* = 1:9). Inset: Absorbance ratio at 499 nm and 462 nm of **P** (20 ppm) upon addition of Cu^2+^ (0–0.6 μM); (**c**) the reversibility experiment: I. **P** (20 ppm), II. **P** (20 ppm) + Cu^2+^ (10 μM), III. **P** (20 ppm) + Cu^2+^ (10 μM) + EDTA (10 μM), IV. **P** (20 ppm) + Cu^2+^ (10 μM) + EDTA (100 μM), V. **P** (20 ppm) + Cu^2+^ (10 μM) + EDTA (100 μM) + Cu^2+^ (10 μM); VI. **P** (20 ppm) + Cu^2+^ (10 μM) + EDTA (100 μM) + Cu^2+^ (100 μM).

Under the same experimental conditions, the interaction of **P** and various Cu^2+^ concentrations were employed to explain the effect on the quenching. As shown in Figure 4a, the gradual increase in Cu^2+^ concentration resulted in a linear decrease in fluorescence intensity, and there was no obvious change in spectral shape. It was found that the quenched fluorescence intensity of **P** was directly proportional to the Cu^2+^ concentration, the emission intensity at 561 nm and Cu^2+^ concentration in the range of 0.5–9 µM, which were found linear with R^2^ = 0.998, indicating that **P** would be a highly efficient fluorescence probe for Cu^2+^; the association constant *K* was determined from the slope to be 1.9 × 10^5^ M^−^^1^, which could be described by a Benesi–Hildebrand equation [24]. Meanwhile, the detection limit of **P** for Cu^2+^ was established at 0.027 µM under current experimental conditions (based on 3 s/*k*, s is the standard deviation of the measured intensity of the blank solution and *k* is the slope of the plot in the inset of Figure 4a), which demonstrated that probe **P** could be utilized for both qualitative and quantitative sensing of Cu^2+^, achieving a sensitivity threshold of 30 µM Cu^2+^ in drinking water according to the World Health Organization (WHO) standard [26]. As seen from Figure 4b, upon sequential addition of Cu^2+^, the absorption band centered at 499 appeared with increasing intensity, which induced a clear color change from pale yellow to orange; meanwhile, the band at 462 nm decreased gradually in intensity, with an isosbestic point at 413 nm. The ratio of absorbance at 499–462 nm increased linearly with the increase in Cu^2+^ concentration (inset of Figure 4b).

Moreover, the fluorescence signal was significantly enhanced by adding different concentrations of EDTA to the solution containing **P** and Cu^2+^ (Figure 4c III–IV), while the fluorescence intensity was again quenched when excessive Cu^2+^ was added (Figure 4c V–VI). The experiment proved that probe **P** had good reversibility, which will be helpful for recycling.

In addition, under the aforementioned optimal experimental conditions, we utilized the standard addition method for quantitative analysis and detection of Cu^2+^ in three types of commercially available bottled water. The results of the analysis are detailed in Table 1. The experimental data indicated a high recovery rate (100.3–118%) for the determination of Cu^2+^ in water samples using this method. Therefore, it is reasonable to infer that fluorescence probe **P** can hold a significant practical application potential for the analysis and detection of Cu^2+^ in real-world samples. Meanwhile, a comparison of Cu^2+^-specific probes is presented in Table 2. Different probes derived from chitosan-based naphthalimide or naphthalimide displayed different characteristics, exhibiting fluorescence enhancement or quenching, which demonstrated a quick response [27,28,29], potential application value [27,30,31,32] and high sensitivity [27,29,31]. Meanwhile, various drawbacks could not be ignored, such as the organic solvent for dissolving [27,28,29,30,31,32,33], the long equilibrium time [31,32], narrow detection ranges [28], and low sensitivity [29,31]. Our probe **P** was a valuable probe with a fast equilibrium time, wide detection range, good reversibility, a visible light for excitation and emission. However, further optimization of probe structure is needed to improve water solubility and extend applications. Generally, **P** had some outstanding superiority to the other mentioned Cu^2+^-probes.

### 3.3. Reaction Mechanism Research

**Figure 5:**
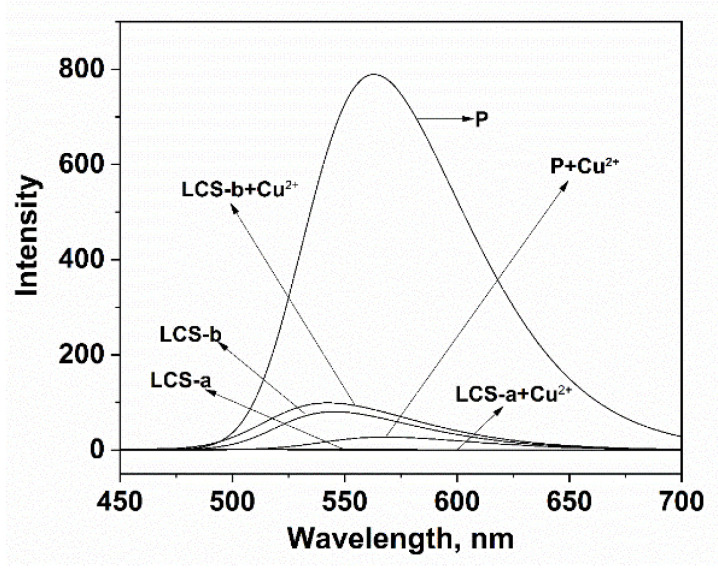
Comparison of selectivity of LCS-a, LCS-b and **P** (20 ppm) for Cu^2+^ (100 µM) in the aqueous-ethanol media (pH 7.0, 20 mM HEPES, *v*:*v* = 1:9).

The probable complexation between **P** and Cu^2+^ was further verified through the selectivity of a series of controls as shown in Figure 5. The experimental results clearly displayed the interaction between each control compound and Cu^2+^. There was no significant fluorescence signal change after Cu^2+^ was added to the solution of LCS-a. The combination of LCS-b and Cu^2+^ only produced a weak fluorescence enhancement at 544 nm. However, when **P** combined with Cu^2+^, the fluorescence quenching phenomenon was obvious at 561 nm, explaining the selectivity of **P** in Cu^2+^ detection and recognition.

### 3.4. Experimental Condition Optimization

**Figure 6:**
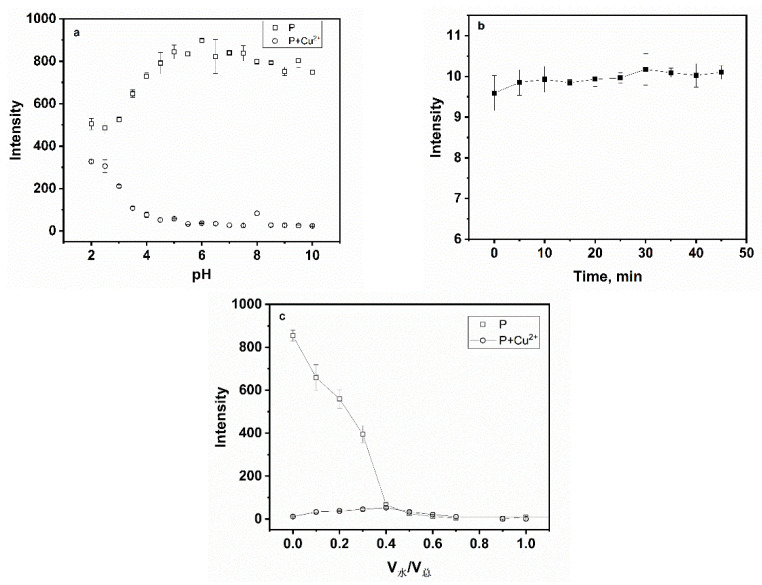
(**a**) Effect of pH on fluorescence spectra of **P** (20 ppm) and **P** (20 ppm) for Cu^2+^ (100 µM); (**b**) Effect of time on the recognition between Cu^2+^ (100 µM) and **P** (20 ppm); (**c**) effect of water content on fluorescence spectra of **P** (20 ppm) and **P** (20 ppm) for Cu^2+^ (100 µM).

The effect of pH was first investigated to evaluate the sensing for Cu^2+^ as depicted in Figure 6a. When pH < 4.5, the fluorescence intensity of **P** at 561 nm increased gradually and a decrease in the fluorescence intensity followed the mixing of **P** with Cu^2+^. When pH > 4.5, the fluorescence intensity of **P** and the **P**-Cu^2+^ system tended to remain fairly static in the wide range of pH 5–10. Thereby, the pH control measurements revealed that **P** was utilized in weak acid, neutral and weakly alkaline environments, which was an advantage in later applications. To study the influence of time on the fluorescence intensity, freshly prepared samples were immediately tested, and then a 5 min interval was set, as seen in Figure 6b. The fluorescence signal was almost completely quenched within 5 min after Cu^2+^ was added to the solution of **P**, which indicated that the reaction between **P** and Cu^2+^ was almost instantaneous. Meanwhile, the effect of water content on fluorescence quenching was also studied. Figure 6c showed that with the increasing volume fraction of water, the fluorescence emission of **P** and **P**-Cu^2+^ can be strongly quenched. In order to further explore the effect between **P** and Cu^2+^, all measurements were carried out in aqueous-ethanol media (pH 7.0, 20 mM HEPES, *v*:*v* = 1:9).

## 4. Conclusions

In summary, we successfully constructed a chitosan-naphthalimide fluorescence probe that is capable of instantaneous selective detection of Cu^2+^. The result showed that the chitosan-based fluorescent probe possessed high selectivity and sensitivity for Cu^2+^ over other common metal ions, which confirmed that our idea was feasible. We believe that this design concept should serve as a reference to develop new chitosan-based probes for transition metal ions.

## Figures and Tables

**Table 1:** Determination of Cu^2+^ in sample water (*n* = 3).

Real Samples	Cu^2+^ (µM)	Sum Results (*n* = 3) (µM)	Recovery (%)
	Added		
Sample 1	6.0	7.0	118
	8.0	8.4	105.4
Sample 2	6.0	6.8	112.7
	8.0	8.0	100.3
Sample 3	6.0	6.9	115.6
	8.0	8.2	102.6

**Table 2:** Performance comparison of various fluorescent probes for Cu^2+^.

Fluorescent Probes	Fluorescence Modes	Respond Time (min)	Reversibility	Linear Range (μM)	LOD (μM)	Testing Media	Applications	Ref.
Naphthalimide derivative	Quench ex/em = 390/520 nm	2	NA	0–7.5	0.0455	Water-DMSO (1:9, *v*:*v*, pH 6.0)	HeLa cells	[27]
Naphthalimide derivative	Quench ex/em = 410/523 nm	2	NA	0.25–4.0	0.015	Water-MeOH (2:1, *v*:*v*, pH 5.5)	NA	[28]
Chitosan-based naphthalimide	Enhancement ex/em = 480/557 nm	1	NA	0–55	4.75	NA	NA	[29]
Naphthalimide derivative	Quench ex/em = 430/525 nm	NA	NA	0.5–5.0	0.567	Water-MeOH (1:1, *v*:*v*, pH 7.4)	River and tap water samples	[30]
Naphthalimide derivative	Enhancement ex/em = 360/432 nm	NA	reversible	0.05–0.9	0.03	Water-EtOH (3:2, *v*:*v*, pH 7.4)	NA	[33]
Chitosan-based naphthalimide	Quench ex/em = 338/479 nm	15	NA	5–100	NA	Acetic acid aqueous solution	Disease diagnose	[31]
Chitosan-based naphthalimide	Quench ex/em = 365/532 nm	30	reversible	0–40	0.029	Water-DMF (6:4, *v*:*v*, pH 7.0)	River, lake and tap water samples	[32]
Chitosan-based naphthalimide	Quench ex/em = 430/561 nm	5	reversible	0.5–9.0	0.027	Water-EtOH (1:9, *v*/*v*, pH 7.0)	NA	This work

## Data Availability

The data presented in this study are available upon request from the corresponding author.

## Supplementary Materials

The following supporting information can be downloaded at: https://www.mdpi.com/article/10.3390/s24113425/s1, Figure S1: ^1^H NMR of LCS-b, Figure S2: ^1^H NMR of **P**.
