# Supplementary material for: Construction of Chitosan-Modified Naphthalimide Fluorescence Probe for Selective Detection of Cu2+
Source: Sensors (Basel). 2024 May 26;24(11):3425. doi: 10.3390/s24113425 (PMC11174907; doi:10.3390/s24113425)
Supplement: Supplementary file 1 [file sensors-24-03425-s001.zip › sensors-3008966-supplementary.pdf]

Supplementary Files

Construction of Chitosan-Modified Naphthalimide Fluorescence Probe for Selective Detection of Cu^{2+}

Chunwei Yu, Jin Huang, Mei Yang and Jun Zhang *

NHC Key Laboratory of Tropical Disease Control, School of Tropical Medicine, Hainan Medical University, Haikou 571199, China; hy0211049@hainmc.edu.cn (C.Y.); abchuangj@163.com (J.H.); yang24364@hainmc.edu.cn (M.Y.)

* Correspondence: jun_zh1979@163.com

1. Figure S1: ^{1}H NMR of **LCS-b**
2. Figure S2: ^{1}H NMR of **P**

Figure S1

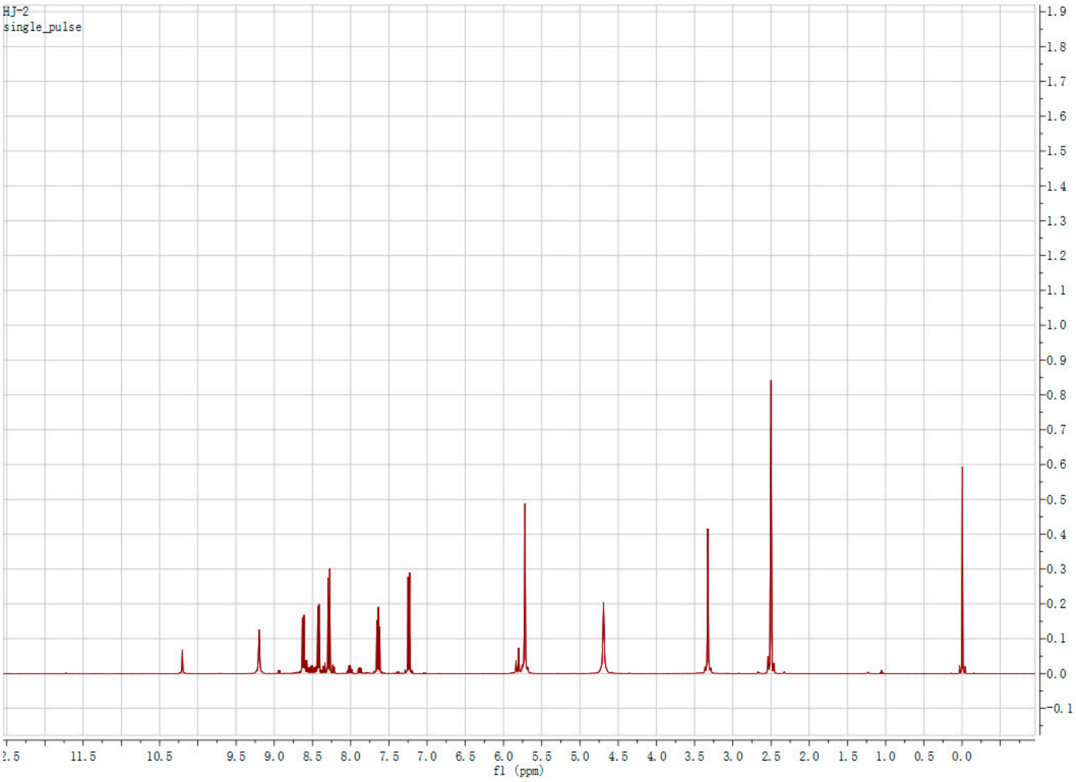

Figure S1 ^{1}H NMR of **LCS-b**

Figure S2

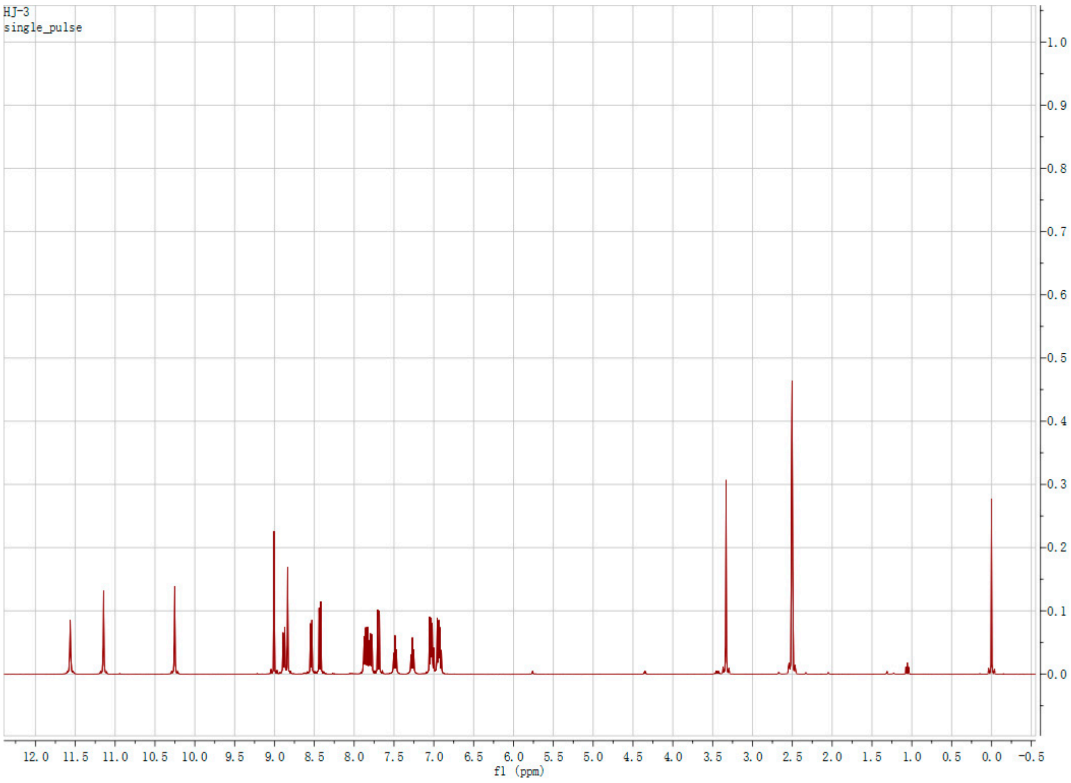

Figure S2 ^{1}H NMR of **P**
